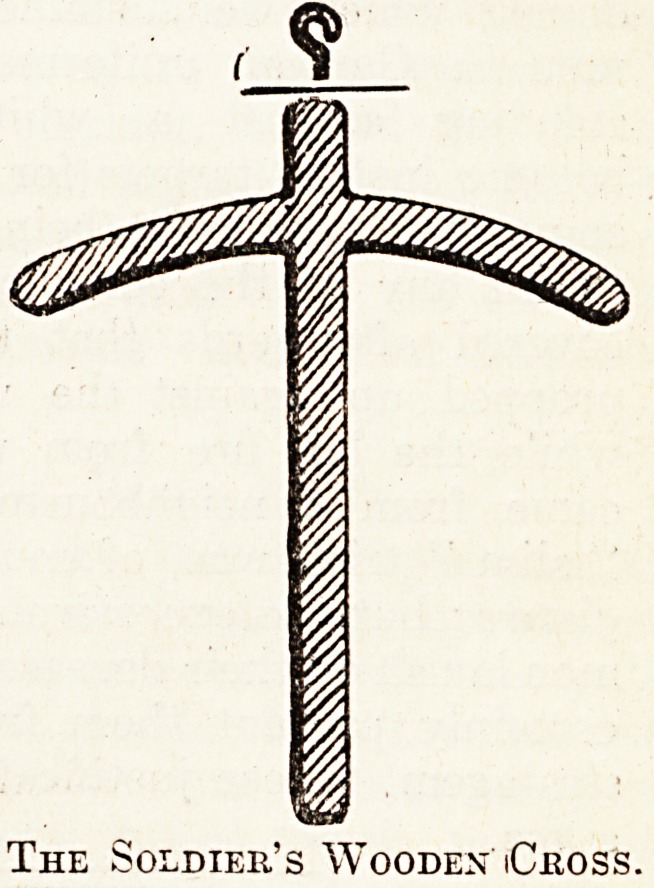# Sidelights upon the War: Reflections and Reminiscences

**Published:** 1914-11-28

**Authors:** 


### SIDELIGHTS UPON THE WAR.

### Reflections and Reminiscences.

BY AN OFFICER OF THE R.A.M.C. AT THE FRONT.

One of the modern improvements introduced at the instance of the Royal Army Medical Corps for the furtherance of hygiene among our troops and the prevention of water-borne disease is the "patent filter" water-cart with which every field unit is supplied. This water-cart requires only a minimum of expert supervision, such as can be (and is) rendered by the R.A.M.C. orderlies attached to each unit for this very purpose, and it delivers at very little trouble a copious supply of filtered and potable water, no matter how suspect may be the source from which the reservoir is filled. This cart is in a considerable degree the outcome of the South African campaign, where water-borne disease proved such a scourge to our army in a country where most of the scanty water supplies were either known to be bad or at least under strong suspicion. Beyond all question this cart, had it been in use at that time, would have saved us hundreds, if not thousands, of casualties, and would have most materially added to the efficiency of the fighting line.

### The Water-carts in France.

In France the conditions have been very different. Water supply has been abundant: sometimes very good in quality, more often moderate, sometimes seriously suspect. The water-cart rendered good service during the hot weather in securing for the troops water which was, with reasonable sureness, free from the micro-organisms of disease. This was of great value at a time when the men were especially thirsty. The campaign has, however, disclosed the limitations of this vehicle as well as its strong points. Most important, the filter water-cart is not nearly strong enough to stand the hard wear and tear of war. It looks strongly built, fit to stand anything; and, indeed, it is strong, but not strong enough. Very few of the filter water-carts which accompanied the Expeditionary Force from England in August are now in use. One after another they have become ineffective through the constant succession of minor injuries and shocks which transport along crowded roads inevitably suffers in war time. Many commanding officers have already left their carts behind as useless encumbrances. Another source of weakness is in regard to the spare "candles" which are issued with the carts, to be fitted while those which may have got clogged are being sterilised and cleaned, or to replace such as may get broken. In practice it is the spare candles which get broken (for lack of sufficient protection) before those in use need restoration. Hence if anything goes wrong with the latter the cart is put out of action for the time being.

There is, however, a bright side to the picture. Water sterilisation by heat in South Africa was impossible by reason of the lack of fuel on the high veld. In France there is abundance of fuel everywhere, and those commanding officers who take a lively interest in hygiene are prohibiting the consumption by their men of any water except that which has been boiled—*e.g.*, by being made into tea. In the trenches this excellent rule may be a counsel of perfection difficult to carry out in its strictest letter. But the fact remains that hitherto the Army has been extraordinarily free from water-borne diseases, notwithstanding the breakdown of the "patent filter" water-cart.

### The Sentimentality of the Soldier.

Thomas Atkins possesses many splendid virtues, and has established them during this war in a manner which extorts the admiration of all beholders. It is not through any lack of appreciation of the magnificent heroism, dogged endurance, and patient fortitude of the soldier that one permits oneself a remark on one of his foibles—namely, an excess of sentimentality. At camp concerts, for example, practically all the songs are morbid to the last degree, full of broken hearts, jilted lovers, meet-me-again-in-heaven, and similar sentimentality of the slushiest kind. The favourite songs are sung to dirge-like tunes so slowly that it seems as if the singer were unwilling to part with each note and was reluctant to let it go completely. The patriotic song, which fairly held its own in South Africa with the sentimental one, is seldom heard in this war; and the rollicking song with a really good rousing chorus is never heard at all unless an officer sings one.

The Soldier's Wooden Cross.

The same sentimentality is exhibited in regard to graves. The soldier, when he has the opportunity, will take endless trouble to make and erect a rough wooden cross over the grave of a fallen comrade, whether one personally known to him or not. This is well enough and deserves nothing but praise; but he sometimes goes further by writing on these crosses the most morbid and gushing inscriptions, thus spoiling the whole effect of an act of simple-hearted piety.

Of late weeks the writer has sometimes observed a method of procuring these monuments which is certainly ingenious. Whether or not it was borrowed from our resourceful foes he cannot say; but it was on German, not English, graves that he first witnessed it. The method consists in appropriating from the cupboard of some abandoned house one of those arc-like wooden frames on which feminine garments are suspended in hanging cupboards. By extracting the hook from the top, a cross with bent arms is obtained, which is utilised to mark the soldier's grave.

Another use to which these dress frames are put —the French cottages and farmhouses contain them in very large numbers—is the manufacture of improvised angular splints for compound fractures about the elbow or in the forearm. One arm is sawn off, also the projecting top piece with the hook, and with the necessary padding the thing is done. The padding, by the way, is frequently hay or straw secured by wrapping the wounded man's puttee round and round the splint.

How the Germans Draw Our Fire.

Nor is the above the only direction in which the soldier displays sentimental traits. The Germans not infrequently draw our fire in the trenches by putting up the bodies of their dead comrades for our men to shoot at. Result: a great waste of ammunition on our part and a disclosure of the exact line held by us. Suggest to Thomas Atkins that he should copy this trick, and he will reject the proposal with great scorn as being an indignity to the dead. On one occasion during an advance by our troops under a hot fire from the enemy, during which we sustained many casualties, five men in German uniforms were seen apparently standing against a whitewashed wall. These became instant targets for our riflemen, who were somewhat puzzled at being (as it seemed) unable to hit any of the conspicuous five. It was discovered afterwards that these were five corpses, propped up against the wall and secured there; while the hot fire from which our men suffered came from a neighbouring cabbage patch. This "slim" trick was, of course, a legitimate *ruse de guerre*; but the excessive sentimentality which our men lavish on their deceased comrades would almost certainly prevent them from copying so useful a stratagem, whose justification is the lives which it saves.

## Figures and Tables

**Figure f1:**